# RURAL RETIREMENT MIGRATION AND ACTIVE CITIZENSHIP: MOTIVATIONS, PRACTICES, AND PROCESSES

**DOI:** 10.1093/geroni/igz038.2401

**Published:** 2019-11-08

**Authors:** Rachel Winterton

**Affiliations:** La Trobe University, Bendigo, Victoria, Australia

## Abstract

Within rural settings, older adults play a significant role in ensuring community age-friendliness through their engagement as active citizens. However, the increasing heterogeneity of rural older adults is challenging expectations around how, and in what circumstances, this cohort will engage as active citizens. Given that rural retirement migration is a key contributor to this increasing heterogeneity, there is a need to understand the motivations for, and practices associated with active citizenship among older in-migrants. Drawing on qualitative data from two rural regions in Victoria, Australia, this paper draws on concepts from the rural citizenship literature to investigate how active citizenship practices of rural retirement migrants align with traditional rural codes of conduct. Semi-structured interviews were conducted with 39 rural retirement migrants (aged 56-76 years), which explored engagement in, and motivations for, active citizenship. Findings indicate that in line with traditional expectations around rural citizenship, most rural retirement migrants had assumed responsibilities associated with community governance, and engaged in protest aimed at defending the rights of rural people. However, conflict with traditional codes of conduct was observed in relation to both how active citizenship was enacted, and motivations for engagement. Additionally, rural retirement migrants highlighted barriers that had precluded their involvement as active citizens. These findings are discussed in relation to their implications for both the capacity of rural settings to meet the needs and expectations of older in-migrants, and the experience of ageing in place for resident older adults.

